# IMPACT OF COVID-19 PANDEMIC ON THE SURGICAL TREATMENT OF GASTRIC CANCER: A 3-YEAR ANALYSIS

**DOI:** 10.1590/0102-6720202400074e1868

**Published:** 2025-01-27

**Authors:** Amanda Juliani ARNEIRO, Marina Alessandra PEREIRA, André Roncon DIAS, Ulysses RIBEIRO, Marcus Fernando Kodama Pertille RAMOS

**Affiliations:** 1 Universidade de São Paulo, Faculdade de Medicina, Hospital das Clínicas, Department of Gastroenterology – São Paulo (SP), Brazil.

**Keywords:** Stomach Neoplasms, Pandemics, COVID-19, Surgical Oncology, Gastrectomy, Neoplasias Gástricas, Pandemias, COVID-19, Oncologia Cirúrgica, Gastrectomia

## Abstract

**BACKGROUND:**

The COVID-19 pandemic has overloaded healthcare systems worldwide. Other diseases, such as neoplasms, including gastric cancer, remained prevalent and had their treatment compromised.

**AIMS:**

The aim of this study was to evaluate the impact of the COVID-19 pandemic on the treatment of gastric cancer and adherence to the recommended preoperative COVID-19 screening protocol.

**METHODS:**

A retrospective study evaluated patients diagnosed with gastric adenocarcinoma who underwent surgical treatment between 2015 and 2023.

**RESULTS:**

A total of 769 patients with gastric cancer were evaluated and organized into two groups: (i) pre-COVID group and (ii) COVID group. The pre-COVID group consisted of 527 patients operated on between 2015 and 2019, and the COVID group consisted of 242 patients from 2020 to 2023. The average number of surgical procedures per year in the pre-COVID group was 105 and 81 in the COVID group. There was a statistically significant difference between ASA classification (p=0.002) and clinical staging (p=0.015), which were worse in the COVID group. We observed an increase in diagnostic surgeries (p=0.026), with an increase in the minimally invasive route (p<0.001). In patients undergoing curative surgery, there was a greater indication for postoperative ICU (p=0.022) and neoadjuvant chemotherapy (p<0.001). There was no difference in 30- and 90-day mortality.

**CONCLUSIONS:**

The surgical and oncological outcomes for patients operated on during the pandemic remained uncompromised, even though many presented with more advanced initial stages and poorer clinical performance. High adherence to protocols and a low rate of complications related to coronavirus indicate that surgeries were performed safely during this period.

## RESUMO

**RACIONAL:**

A pandemia de COVID-19 levou a sobrecarga dos sistemas de saúde em todo o mundo. Outras doenças como as neoplasias, dentre elas o câncer gástrico, continuaram prevalentes e tiveram seu tratamento comprometido.

**OBJETIVOS:**

Avaliar o impacto da pandemia de COVID-19 no tratamento do câncer gástrico e a adesão ao protocolo de triagem pré-operatória de COVID-19 recomendado.

**MÉTODOS:**

Estudo retrospectivo que avaliou pacientes com diagnóstico de adenocarcinoma gástrico submetidos a tratamento cirúrgico no período 2015 a 2023.

**RESULTADOS:**

Foram avaliados 769 pacientes com câncer gástrico. O Grupo Pré-COVID, foi composto por 527 pacientes operados entre 2015 e 2019 e Grupo COVID, formado por 242 pacientes de 2020 a 2023. A média de procedimentos cirúrgicos por ano no Grupo Pré-COVID foi de 105 e 81 no Grupo COVID. Houve diferença estatisticamente significativa entre Classificação ASA (p=0,002) e estadiamento clínico (p=0,015), piores no Grupo COVID. Observamos aumento de cirurgias diagnósticas (p=0,026), com aumento da via minimamente invasiva (p<0,001). Nos pacientes submetidos a cirurgia curativa, observa-se maior indicação de UTI pós-operatória (p=0,022) e quimioterapia neoadjuvante (p<0,001). Não houve diferença na mortalidade 30 e 90 dias.

**CONCLUSÕES:**

Não houve comprometimento dos resultados cirúrgicos e oncológicos dos pacientes operados durante a pandemia, apesar do estádio inicial mais avançado e pior performance clínica. A boa adesão ao protocolo e a baixa taxa de complicações pelo coronavírus demonstram que houve segurança na realização das cirurgias no período.

## INTRODUCTION

The disease known as COVID-19 was first detected in December 2019 in Wuhan, China, and rapidly spread worldwide due to its high transmissibility. In March 2020, the World Health Organization (WHO) declared COVID-19 a pandemic, causing more than six million deaths worldwide since then. The spread of COVID-19 affected populations worldwide across various sectors, notably impacting socioeconomic and healthcare systems. The pandemic triggered a public health and hospital crisis, limiting access for patients with other conditions to healthcare services^2,11,12^.

Other diseases, such as cancer, faced diagnostic and treatment delays as resources shifted to prioritize COVID-19 patients. Studies have shown that oncology patients are at an increased risk for severe COVID-19 infection and subsequent complications, especially when undergoing surgery or chemotherapy in the month preceding COVID-19 infection. A postoperative diagnosis of COVID-19 has been associated with a higher need for ICU admission and an elevated risk of mortality, compared to COVID-19 patients without a prior cancer diagnosis^18^.

Among malignancies, gastric cancer (GC) ranks as the fifth most common cancer in Brazil and holds the third position in cancer-related mortality^21^. Due to its high prevalence, GC diagnosis may have been particularly impacted during the pandemic, largely due to reduced endoscopic procedures and decreased hospital attendance^19,21^.

Globally and at our Institution, the Cancer Institute of the Hospital das Clinicas in São Paulo-Brazil (ICESP), preventive measures were implemented to minimize COVID-19 transmission from admission to discharge. A triage protocol was established, incorporating increased teleconsultations, preoperative RT-PCR testing via nasopharyngeal swabs, and visitor restrictions for inpatients. Elective surgeries were postponed, prioritizing operations for patients with more advanced cancer, while surgeries for those with early stage, potentially deferrable cancer were postponed early in the pandemic.

Our 2021 study, also conducted at ICESP, assessed the impact of the first pandemic year, showing a reduction in the average number of surgeries and a higher frequency of diagnostic procedures. However, there was no increase in morbidity rates, and mortality rates in GC patients during the first pandemic year did not differ from pre-pandemic levels^3^.

However, the broader influence of pandemic-related restrictions on GC treatment outcomes remains unknown. Therefore, the present study aimed to evaluate the impact of COVID-19 on surgical treatment and clinicopathological characteristics of GC patients over the 3 years of the pandemic. Additionally, the preoperative COVID-19 triage protocol adopted by the institution during this period was also assessed.

## METHODS

Patients diagnosed with gastric adenocarcinoma who underwent surgical treatment between 2015 and 2023 at the Cancer Institute of the Hospital das Clinicas in São Paulo (ICESP) were evaluated retrospectively. Patients who had undergone gastric surgeries as part of treatment for another primary tumor or for non-gastric cancer-related procedures were excluded. The patients were divided into two groups: the pre-COVID group, consisting of patients who underwent surgeries between 2015 and 2019, and the COVID group, including patients operated on from March 2020 to March 2023.

Initial clinical characteristics assessed in the patients included age, sex, weight, body mass index (BMI), serum albumin, hemoglobin, neutrophil-to-lymphocyte ratio, presence of comorbidities (Charlson Comorbidity Index), American Society of Anesthesiologists Classification (ASA), and Laurén histological type. The study outcomes included the surgical treatment indication (diagnostic, curative, or palliative), surgical access route (open or minimally invasive), postoperative ICU use, surgical complications (Clavien-Dindo classification), length of hospital stay, surgical mortality, TNM stage (8th edition), chemotherapy, preoperative COVID-19 screening, and postoperative COVID-19 infection^6,9^.

The data were organized as mean and standard deviation (±SD), median and interquartile range (IQR) for continuous variables, and as absolute numbers and percentages for categorical data. Group comparisons were performed using Pearson’s chi-square test or Fisher’s exact test for categorical variables, Student’s t-test or Mann–Whitney U test (non-parametric) for quantitative variables, and ANOVA or Kruskal-Wallis test (non-parametric) for comparisons across more than two groups.

Statistical analyses were performed using SPSS version 20.0 (SPSS Inc., Chicago, IL). Results were considered significant at p<0.05. The study received approval from the hospital’s Ethics Committee and was registered on the Brazil Platform (CAAE number 44352421.2.0000.0068). An exemption was granted for the requirement of a signed informed consent form.

## RESULTS

Between 2015 and 2023, a total of 769 patients with GC underwent surgical treatment at ICESP. These patients were divided into two groups: the pre-COVID group, with 527 patients who had surgeries between 2015 and 2019, and the COVID group, consisting of 242 patients operated on from March 2020 to March 2023.

**Figure 1:**
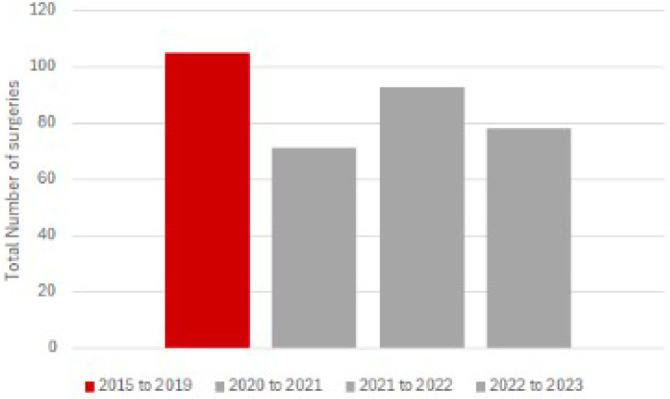
Average annual surgical procedures for the treatment of gastric cancer in the pre-COVID group and COVID group.

The mean number of surgical procedures per year in the pre-COVID group was 105. In the COVID group, the mean was 81 procedures, with 71 cases in the first year (2020–2021), 93 cases in the second (2021–2022), and 78 in the final year (2022–2023), as shown in Figure 1.

Table 1 presents the clinical, surgical, and pathological characteristics of all patients in the pre-COVID and COVID groups. No significant differences were observed between the groups regarding sex, age, BMI, and comorbidities. There was a statistically significant difference in ASA classification (p=0.002) and clinical staging (p=0.015). Statistically significant differences were also found in the type of surgery (p=0.026) and surgical access route, with a higher prevalence of minimally invasive procedures (p<0.001) and in the administration of preoperative (p<0,001) and postoperative chemotherapy (p=0.024). There was no difference in hospital length of stay or 30- and 90-day survival rates between the two groups.

**Table 1:** Clinical, surgical, and pathological characteristics of all patients in the pre-COVID and COVID groups.

Variables	Group		p-value
	Pre-COVID	COVID	
	n=527 (%)	n=242 (%)	
Sex			
Female	197 (37.4)	99 (40.9)	0.350
Male	330 (62.6)	143 (59.1)	
Age (years)			
Mean (SD)	62.2 (12.8)	62.0 (11.9)	0.788
cT			
cT1/T2	179 (34)	61 (25.2)	0.015
cT3/T4	348 (66)	181 (74.8)	
cN			
cN0	169 (32.1)	79 (32.6)	0.874
cN+	358 (67.9)	163 (67.4)	
cM			
cM0	383 (72.7)	180 (74.4)	0.620
Type of surgery			
Curative	336 (63.8)	141 (58.3)	0.026
Palliative	127 (24.1)	58 (24)	
Diagnostic	48 (9.1)	39 (16)	
Conversion to open surgery	16 (3)	4 (1.7)	
Access			
Conventional	383 (72.7)	144 (59.5)	<0.001
Laparoscopic	144 (27.3)	98 (40.5)	
Preoperative chemotherapy			
No	460 (87.3)	173 (71.5)	<0.001
Yes	67 (12.7)	69 (28.5)	
Postoperative chemotherapy			
No	380 (72.1)	193 (79.8)	0.024
Yes	147 (27.9)	49 (20.2)	

SD: standard deviation; T: Tumor; N: Lymph node; M: metastasis.

Table 2 presents the clinical and surgical characteristics of patients in the pre-COVID and COVID groups who underwent surgical resection with curative intent. A statistical difference was observed between the groups in ASA classification (p=0.002), postoperative ICU admission rate (p=0.022), and the use of neoadjuvant chemotherapy (p<0.001).

**Table 2:** Clinical and surgical characteristics of patients undergoing surgical resection with curative intent in the pre-COVID and COVID groups.

Variables	Group		p-value
	Pre-COVID	COVID	
	n=334 (%)	n=141 (%)	
Sex			
Female	133 (39.8)	60 (42.6)	0.580
Male	201 (60.2)	81 (57.4)	
Age (years)			
Mean (SD)	62.7 (13.0)	63.3 (11.0)	0.400
Body max index (kg/m^2^)			
Mean (SD)	24.6 (4.6)	24.8 (5.3)	0.781
Charlson-Deyo Comorbidity Index			
0	219 (65.6)	89 (63.1)	0.610
≥1	115 (34.4)	52 (36.9)	
American Society of Anesthesiologists			
I/II	224 (67.1)	73 (51.8)	0.002
III/IV	110 (32.9)	68 (48.2)	
Postoperative ICU			
No	188 (68.1)	74 (56.5)	0.022
Yes	88 (31.9)	57 (43.5)	
Preoperative chemotherapy			
No	289 (86.5)	95 (67.4)	<0.001
Yes	45 (13.5)	46 (32.6)	
Mortality (days)			
30	8 (2.4)	7 (5)	0.157
90	24 (7.2)	10 (7.1)	0.971
Length of hospital stay (days)			
Mean (SD)	13.6 (12.0)	13.9 (14.7)	0.839

ICU: intensive care unit; SD: standard deviation.

Table 3 shows the pathological characteristics of patients in the pre-COVID and COVID groups who underwent surgical resection with curative intent. There was no significant statistical difference in lesion location, Laurén histological type, or differentiation grade. However, the COVID group had a higher frequency of venous invasion compared to the pre-COVID group (p=0.021).

**Table 3:** Pathological characteristics of patients undergoing surgical resection with curative intent in the pre-COVID and COVID groups.

Variable	Group		p-value
	Pre-COVID	COVID	
	n=334 (%)	n=141 (%)	
Location of the tumor			
Distal	204 (61.1)	79 (56)	0.627
Medial	94 (28.1)	42 (29.8)	
Proximal	34 (10.2)	19 (13.5)	
Diffuse (linite)	2 (0.6)	1 (0.7)	
Lauren`s histological type			
Intestinal	188 (56.3)	69 (48.9)	0.238
Diffuse/mixed	141 (42.2)	71 (50.4)	
Not specified	5 (1.5)	1 (0.7)	
Tumor differentiation			
G1/G2	158 (47.3)	63 (44.7)	0.862
G3	172 (51.5)	77 (54.6)	
Not specified	4 (1.2)	1 (0.7)	
Invasion			
Lymphatic	158 (48.2)	70 (50)	0.717
Venous	113 (34.5)	64 (45.7)	0.021
Perineural	152 (46.3)	65 (46.4)	0.986
pT			
pT1/pT2	145 (43.4)	67 (47.5)	0.411
pT3/pT4	189 (56.6)	74 (52.5)	
pN			
pN0	154 (46.1)	71 (50.4)	0.368
pN+	180 (53.9)	70 (49.6)	

G: grade; T: tumor; N: lymph node.

To evaluate the results over the years of the pandemic, patients were separately evaluated from 2020 to 2023. When considering only curative procedures within the COVID group, 40 patients were operated on in the first year, 58 in the second, and 43 in the third year of the pandemic.

Table 4 presents the clinical, surgical, pathological characteristics, and early outcomes of GC patients undergoing curative treatment per year in the COVID group. There was no statistical difference in clinical characteristics such as sex, age, BMI, and comorbidities among the groups. Differences were observed in ASA classification (p=0.029) and neutrophil-to-lymphocyte ratio (p=0.02).

**Table 4:** Clinical, pathological, surgical characteristics, and early outcomes of patients with gastric cancer operated per year in the COVID group with curative treatment.

Variables	COVID - Curative			p-value
	Year 1	Year 2	Year 3	
	n%=40	n%=58	n%=43	
Neutrophil-lymphocyte ratio				
Mean (SD)	3.81 (4.93)	2.59 (2.05)	5.49 (6.21)	0.020
Charlson-Deyo comorbidity index				
0	23 (57.5)	40 (69)	26 (60.5)	0.467
≥1	17 (42.5)	18 (31)	17 (39.5)	
American Society of Anesthesiologists				
ASA I/II	24 (60)	34 (58.6)	15 (34.9)	0.029
ASA III/IV	16 (40)	24 (41.4)	28 (65.1)	
Postoperative ICU				
No	26 (65)	34 (58.6)	14 (42.4)	0.139
Yes	14 (35)	24 (41.4)	19 (57.6)	
Preoperative chemotherapy				
No	30 (75)	38 (65.5)	27 (62.8)	0.458
Yes	10 (25)	20 (34.5)	16 (37.2)	
Mortality (days)				
30	1 (2.5)	3 (5.2)	2 (4.7)	0.720
90	3 (7.5)	3 (5.2)	4 (9.3)	0.713
Length of hospital stay (days)				
Mean (SD)	13 (13.1)	14 (15.8)	15 (14.9)	0.935

SD: standard deviation; ICU: intensive care unit.

**Figure 2:**
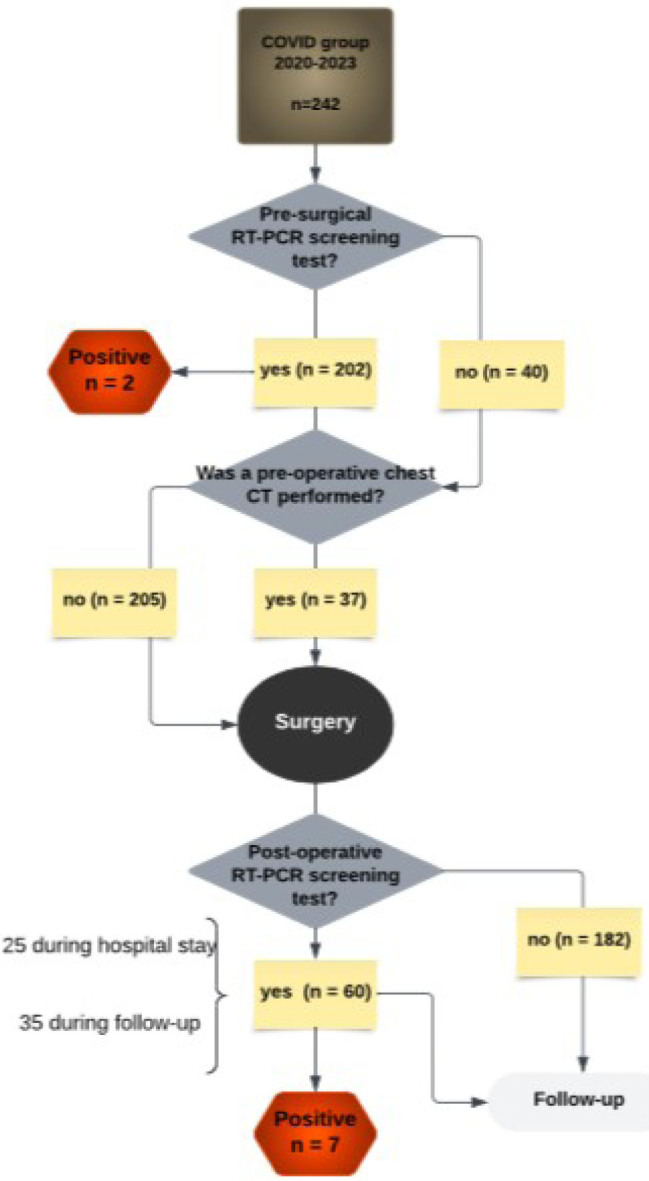
Results of screening for COVID-19 infection in gastric cancer patients undergoing surgery.

During the 3-year pandemic period, 40 (16.5%) patients were not tested preoperatively. Among the 202 patients tested with RT-PCR, 2 tested positive for COVID-19 and had their procedures postponed. For chest computed tomography, screening scans were conducted on 37 patients. The remaining patients had staging chest CT scans and did not undergo additional imaging. During the postoperative period, 60 patients required COVID-19 testing, 25 during hospitalization, and the rest during follow-up. Among those tested, seven patients were positive for COVID-19, as shown in Figure 2. There were no COVID-19-related deaths during the study period.

## DISCUSSION

Following global trends, a reduction in the annual average of surgeries was observed during the pandemic, although this difference was not statistically significant. There was a significant worsening in both ASA classification and clinical staging among patients operated on throughout the pandemic period. Additionally, there was an increase in the use of minimally invasive techniques, diagnostic surgeries, and preoperative chemotherapy. These findings suggest a progressive decline in the clinical status of oncology patients, reflecting the adverse and cumulative impacts of the healthcare crisis on the surgical and therapeutic management of these patients.

Although no significant difference was observed, there was a reduction in the absolute number of procedures, which may indicate a trend likely to reach statistical significance over time. Within the Hospital das Clínicas complex, the Cancer Institute was designated as a COVID-19-free zone and maintained its operations, albeit on a smaller scale, during the pandemic. As a result, the Institute could continue performing elective oncological surgeries alongside some urgent non-oncological procedures. In this context, gastrectomy for the treatment of GC remained a priority during the pandemic, as it generally requires less postoperative ICU use compared to other surgeries for gastrointestinal neoplasms. The indication for ICU care after gastrectomies is more often due to patient comorbidities than the surgical complexity itself.

Globally, the overall reduction in surgeries has also been attributed to other factors, such as delays in seeking medical care and diagnosis, resulting from limited access to outpatient consultations and hospital facilities^10,23^. Regarding GC, this decrease in surgeries may reflect the reduced rate of cancer diagnoses during the pandemic. This phenomenon is largely attributed to the reduction in endoscopic examinations, considered the gold standard for diagnosis, which were limited due to concerns over aerosol exposure^5,13,14,25^.

In addition to the decrease in diagnosis and number of surgeries performed during the pandemic, the present study demonstrated a trend of worsening clinical conditions in operated patients, as evidenced by their ASA classification. The COVID group included patients with higher ASA classifications (III/IV), indicating increased risk due to poorer overall performance status. This trend was also observed in patients undergoing curative surgeries as the pandemic progressed, suggesting that by the third year of the pandemic, the patients undergoing surgery were in worse clinical condition. These findings indicate that the pandemic affected not only the number of procedures performed but also the severity of clinical conditions in operated patients. Additionally, patients seeking medical care during the pandemic were likely more symptomatic or debilitated, thus more willing to take the risks associated with seeking surgical treatment.

However, despite differences in ASA classification, there was no significant statistical difference in the Charlson-Deyo score between the periods. This indicates that these patients did not differ in the number of comorbidities. Instead, they exhibited greater severity of pre-existing comorbidities or increased clinical impairment due to GC.

Another indicator of the patient’s worsening clinical status throughout the pandemic was the progressive increase in the neutrophil-to-lymphocyte ratio (NLR) each year. Elevated NLR is recognized as an independent predictor of poor prognosis, correlating with decreased overall survival in GC patients undergoing curative gastrectomy^24^.

Clinical staging also worsened throughout the pandemic, with a significant increase in T3/T4 staging observed in the COVID group compared to the pre-COVID group. This finding aligns with global studies, which have similarly reported a higher proportion of advanced tumors during the pandemic period^16,22^.

In the present study, an increase in the number of diagnostic surgeries and minimally invasive procedures was observed, alongside a rise in the use of preoperative chemotherapy. Preoperative chemotherapy gained prominence following the publication of the MAGIC trial in 2006^8^, which highlighted its benefits. The rationale for its use included improved tolerance to chemotherapy when administered before surgery, the potential for tumor downstaging, and enhanced survival outcomes. Initial studies on neoadjuvant chemotherapy faced criticism due to the inclusion of a high number of patients with distal esophageal tumors and suboptimal lymphadenectomy quality. However, in 2019, the German FLOT-4 study demonstrated superior pathological and survival outcomes without increasing perioperative complications^1^. Since then, preoperative chemotherapy has been recommended for patients with cT3/T4 staging or suspected lymph node involvement^4,7^. The timing of this publication proved critical, as it facilitated greater adoption of preoperative chemotherapy as a strategy to optimize patient management during a period of limited resources. It allowed patients to continue receiving treatment while surgical safety was being reassessed in the early stages of the pandemic.

With the increased adoption of neoadjuvant chemotherapy, a rise in minimally invasive surgeries was anticipated, driven by the recommendation for diagnostic laparoscopy. Currently, diagnostic laparoscopy is advised for all patients with advanced GC to identify occult peritoneal metastases, which can occur in up to 30% of cases^20^.

The reduction in the number of surgeries, the increase in patients with higher ASA classifications, and the rise in T3/T4 staging likely contributed to the observed increase in ICU admissions during the postoperative period in this study. However, no difference in patient length of stay was noted. This finding contrasts with other studies, which reported longer postoperative hospital stays in some countries during the pandemic^15,17^.

Despite presenting with more compromised clinical status and more advanced TNM clinical staging, no increase in 30- or 90-day mortality was observed. This indicates that there was no heightened risk of patients acquiring infections or experiencing higher mortality during hospitalization, underscoring the safety of continuing surgeries during the pandemic. These findings suggest that, despite the challenges faced, postoperative management was effective in preventing significant extensions of hospital stays. This highlights the institution’s adaptability in maintaining high-quality care for oncological patients even under adverse conditions.

Regarding the COVID-19 screening protocol, a high adherence rate was observed. Among the tested patients, none returned a positive result preoperatively. Preoperative testing was crucial to mitigate the risks of operating on infected patients, who might experience complicated postoperative outcomes, and to prevent exposing a designated COVID-free hospital environment to infected individuals. Among the patients tested postoperatively, only seven had positive COVID-19 results, with no associated severe complications. Additionally, there were no COVID-19-related deaths among the evaluated patients. These results further demonstrate the safety and effectiveness of maintaining surgical care during the pandemic, with no increased risk of infection or mortality observed during hospitalization.

This study has some limitations. First, as only hospital-based data were analyzed, it was not possible to assess the time interval between the initial diagnostic examination conducted at the primary healthcare unit and the referral for treatment at ICESP. This temporal gap could have a significant impact on treatment outcomes^10^. Additionally, patients referred exclusively for palliative chemotherapy or those planned for preoperative chemotherapy but who did not undergo surgery were not evaluated. If any of these patients died during chemotherapy, whether related to COVID-19 infection or not, they would not have been identified due to the absence of a surgical procedure. Furthermore, the lack of information regarding immunization status, including whether patients had completed the COVID-19 vaccination schedule, is another limitation that is worth acknowledgment.

Despite these limitations, the results demonstrated no significant changes in short-term surgical or oncological outcomes. This finding is crucial as it reaffirms the ability of tertiary care centers to maintain high-quality oncological care despite the challenges posed by the global health crisis. It also highlights the importance of ensuring the continuity of cancer treatments even during periods of immense pressure on the healthcare system.

## CONCLUSIONS

There were no differences in surgical or oncological outcomes between patients who operated during the pandemic and those who operated before it. An increase in diagnostic surgeries, perioperative chemotherapy, and postoperative ICU use was observed during the pandemic. The high adherence to protocols, low complication rates, and absence of mortality during the period demonstrate the safety of performing and recommending surgical procedures amid the pandemic.
